# Syndrome d'Eagle à propos d'un cas

**DOI:** 10.11604/pamj.2014.18.333.5192

**Published:** 2014-08-26

**Authors:** Faiçal Choumi, Yassine Ziani

**Affiliations:** 1 Service de Chirurgie Plastique, Maxillo-Faciale et Stomatologie, Hôpital Militaire Moulay Ismail, Meknes, Maroc; 2 Service de Chirurgie Plastique, Maxillo-Faciale et Stomatologie, Hôpital Militaire d'Instruction Mohamed V, Rabat, Maroc

**Keywords:** Syndrome d'Eagle, ligament stylo-hyoïdien, cervicalgies, Eagle Syndrome, stylohyoid ligament, neck pain

## Image en medicine

Le syndrome d'Eagle est une entité radio-clinique caractérisée par une ossification du ligament stylo-hyoïdien qui peut se manifester par des signes cliniques en rapport avec la compression de structures vasculo-nerveuses de voisinage. Nous rapportons un cas de syndrome d'Eagle de découverte fortuite chez une patiente de 62ans, sans antécédent particulier, admise aux urgences pour une fracture de l'arcade zygomatique droite à la suite d'une chute. La tomodensitométrie(TDM) maxillo-faciale avec reconstruction 3D a objectivé deux processus osseux bilatéraux s’étendant de l'apophyse styloïde en direction de la petite corne de l'os hyoïde témoignant d'une ossification du ligament stylo-hyoïdien, ce qui a permis de poser le diagnostic de syndrome d'Eagle. Le syndrome d'Eagle ou syndrome de l'apophyse styloïde longue a été décrit pour la première fois par Eagle en 1937. Sa fréquence est estimée à 4% de la population générale, et seulement 4% de ses ossifications sont symptomatiques. Les signes fonctionnels sont variables, Eagle a distingué trois groupes, le premier est celui du syndrome classique associant des cervicalgies, otalgie et une gêne pharyngée, le deuxième caractérisé par des douleurs le long de la carotide externe et le troisième asymptomatique. La palpation des fossettes tonsillaires permet de suspecter le diagnostic. Les radiographies standards le confirment généralement, mais c'est surtout la TDM qui permet de bien explorer le ligament calcifié et ses rapports. Le traitement est chirurgical, basé sur l'exérèse du processus calcifié et la libération des structures vasculo-nerveuses comprimées. Une infiltration locale de corticoïdes pourra être proposée pour les patients peu symptomatiques.

**Figure 1:**
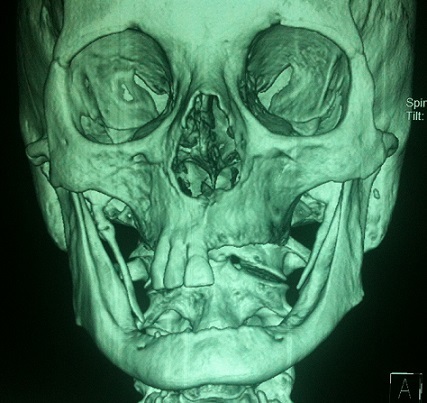
TDM maxillo-faciale avec reconstruction en 3D montrant deux processus osseux bilatéraux s’étendant de l'apophyse styloïde en direction de la petite corne de l'os hyoïde témoignant d'une ossification du ligament stylo-hyoïdien

